# The Proposed Scotch Ascendancy in the College of Physicians

**Published:** 1834-01-01

**Authors:** 


### MEDICAL POLITICS AND INTELLIGENCE.

### I. THE PROPOSED SCOTCH ASCENDANCY IN THE COLLEGE OF PHYSICIANS.

There is a pleasant story in the books which bears upon its face every mark of authenticity. An Irishman, delighted with the flavour of an apple-pie into which the cook had put a quince or two, declared that there was nothing which he should like so much, as an apple-pie made entirely of quinces. Now this story, besides its intrinsic merits, obviously allegorises the popular feeling with respect to the distribution of honours. Let us, cry the mob, have no invidious distinctions; in the great pie of society, let every apple be a quince. Suppose the prayer of the mob to be granted, what is the consequence? The gustatory nerves are first over-stimulated, and then lulled into torpor; or, to drop the metaphor, the respect paid to the new honours soon gives place to indifference or contempt. To illustrate this fact, it is not necessary to leave our own country, or to inquire whether titles of honour have attained their minimum of value in France, Italy, or Germany; we can find instances at home. To be an esquire was once a considerable distinction; it is now bestowed with a lavish hand by every one who can write a letter: to be an M.D. formerly signified that a man was qualified not merely to practise physic but to teach it; it now very often means merely that he is in possession of a piece of parchment, sent him by some unheard-of university.

Another honour is now to be defaced, to lose its freshness and its bloom. Let us in, cries the invading army of Aberdeen; and it is to be feared that the demand will be assented to, and the garrison will march out — without any of the honours of war. At present, the Fellowship of the College of Physicians is a desirable distinction; throw it open to all that ask for it, and they gain little but a short-lived triumph, while we have lost the commodious link which connects the heads of the profession with the gentlemen of England. In refusing to grant this title indiscriminately, we would not therefore deny it to all; but some measure is to be observed in this kind of generosity. When St. Martin gave half his cloak to a frozen beggar, he was a charitable saint; had he divided it among a thousand, he had been but a crazed humourist. We would propose that the licentiates, without limitation as to number, should be eligible as fellows, after passing ten years in their probationary state. To concede much more than this would be weak, to concede much less might seem illiberal. It would surely be unreasonable for the holder of a Scotch diploma to obtain everything at once; it is as if the possessor of a Columbian bond were to expect to be paid in full. Men do not gather figs from thistles, and the half-honours of Aberdeen or St. Andrew's would be rather over than undervalued in our benevolent scheme.

